# Use of extracorporeal membrane oxygenation in Croatian lung transplantation programme. Our initial experience

**DOI:** 10.1186/1749-8090-8-S1-P34

**Published:** 2013-09-11

**Authors:** G Pavlisa, H Puretic, E Zuljevic, M Jakopovic, G Redzepi, M Jankovic, V Ivancan, B Biocina, M Samarzija

**Affiliations:** 1 Department of Pulmonary Medicine, University Hospital Center Zagreb, Zagreb, Croatia; 2 Department of Anesthesiology, Reanimatology and Intensive Care Medicine, University Hospital Center Zagreb, Zagreb, Croatia; 3 Department of Cardiac Surgery, University Hospital Center Zagreb, Zagreb, Croatia

## 

Extracorporeal membrane oxygenation (ECMO) is indicated in refractory respiratory or cardiac failure. Despite its' effectiveness, many centers consider ECMO as inappropriate for bridging to lung transplantation because of poor outcomes. We present three consecutive patients treated by ECMO as a bridging strategy to lung transplantation.

## Case 1

A 52-year-old female with the history of idiopathic pulmonary fibrosis was hospitalized due to complete right-sided pneumothorax. Despite the invasive mechanical ventilation and thoracic drainage with continuous suction, severe respiratory failure persisted and veno-venous ECMO was initiated. On a second ECMO day, a suitable donor for transplantation became available. She was transferred to the transplantation center in Vienna by the air service using transportable ECMO device and underwent double lung transplantation. She remains stable in two years follow-up.

## Case 2

A 55-year-old female presented with severe hypoxaemic respiratory failure due to lung sarcoidosis stage IV, which was previously untreated. She was placed on veno-venous ECMO because of progressive respiratory failure despite immunosuppressive therapy. She developed pneumonia with multi-resistant Acinetobacter baumanii and Aspergillus fumigatus which were a contraindication for transplantation. In spite of treatment she died of sepsis and multiorgan failure after 57 days of ECMO.

## Case 3

A 60-year-old male with chronic obstructive pulmonary disease and severe pulmonary hypertension scheduled for lung transplantation presented with acute right ventricular failure which required institution of veno-arterial ECMO. On a second ECMO day, he underwent double lung transplantation. The procedure was uneventful, and he has been stable in one year follow-up.

ECMO could be an effective method of bridging patients to lung transplantation. It enables sufficient respiratory and circulatory support during the transport to the referral center.

